# Thermophilic bacteria in Moroccan hot springs, salt marshes and desert soils

**DOI:** 10.1590/S1517-838246220140219

**Published:** 2015-06-01

**Authors:** Tarik Aanniz, Mouna Ouadghiri, Marouane Melloul, Jean Swings, Elmostafa Elfahime, Jamal Ibijbijen, Mohamed Ismaili, Mohamed Amar

**Affiliations:** 1 Laboratoire de Microbiologie et Biologie Moléculaire, Centre National pour la Recherche Scientifique et Technique, Rabat, Maroc, Laboratoire de Microbiologie et Biologie Moléculaire, Centre National pour la Recherche Scientifique et Technique, Rabat, Maroc.; 2 Laboratoire de Microbiologie et Biologie Moléculaire, Centre National pour la Recherche Scientifique et Technique, Rabat, Maroc, Collections Coordonnées Marocaines de Microorganismes, Laboratoire de Microbiologie et Biologie Moléculaire, Centre National pour la Recherche Scientifique et Technique, Rabat, Maroc.; 3 Faculté des Sciences, Université Moulay Ismail, Meknès, Maroc, Faculté des Sciences, Université Moulay Ismail, Meknès, Maroc.; 4 Laboratory of Microbiology, Gent University, Gent, Belgium, Laboratory of Microbiology, Gent University, Gent, Belgium.; 5 Unité d'Appui Technique à la Recherche Scientifique, Centre National pour la Recherche Scientifique et Technique, Rabat, Maroc, Unité d'Appui Technique à la Recherche Scientifique, Centre National pour la Recherche Scientifique et Technique, Rabat, Maroc.

**Keywords:** thermophilic bacteria, hot springs, diversity, 16S rRNA gene sequencing, hydrolytic enzymes

## Abstract

The diversity of thermophilic bacteria was investigated in four hot springs, three salt marshes and 12 desert sites in Morocco. Two hundred and forty (240) thermophilic bacteria were recovered, identified and characterized. All isolates were Gram positive, rod-shaped, spore forming and halotolerant. Based on BOXA1R-PCR and 16S rRNA gene sequencing, the recovered isolates were dominated by the genus *Bacillus* (97.5%) represented by *B. licheniformis* (119), *B. aerius* (44), *B. sonorensis* (33), *B. subtilis* (subsp*. spizizenii* (2) and subsp*. inaquosurum* (6)), *B. amyloliquefaciens* (subsp*. amyloliquefaciens* (4) and subsp*. plantarum* (4)), *B. tequilensis* (3), *B. pumilus* (3) and *Bacillus* sp. (19). Only six isolates (2.5%) belonged to the genus *Aeribacillus* represented by *A. pallidus* (4) and *Aeribacillus* sp. (2). In this study, *B. aerius* and *B. tequilensis* are described for the first time as thermophilic bacteria. Moreover, 71.25%, 50.41% and 5.41% of total strains exhibited high amylolytic, proteolytic or cellulolytic activity respectively.

## Introduction

Extremophiles are thriving in extreme ecosystems. Such environments may have extremely high or low pH, high or low temperatures, high salinity, high pressure and various combinations thereof. Extremophilic microorganisms include members of all three domains of life, Archaea, Bacteria and Eukarya. Many investigations focused on their potential as sources of highly active enzymes ‘extremozymes' and other products such as antibiotics, compatible solutes (Robb *et al.*, 2008).

Thermophiles are growing optimally between 55 and 80 °C, while hyperthermophiles grow above 80 °C (Brock 1978; Bertoldo *et al.*, 2002). They may be Gram positive or negative, spore forming or not, and may exhibit an aerobic or anaerobic metabolism. Their study has become a major domain of research and several new thermophilic genera and species have recently been described (Yoneda *et al.*, 2013; Cihan *et al.*, 2014). They were intensively studied due to their potential to produce thermostable enzymes (proteases, amylases, lipases, xylanases, DNA polymerases) and exo-polysaccharides (Meintanis *et al.*, 2008; Singh *et al.*, 2010). These thermo-enzymes are usually not only thermostable, but also active at high salinity and extreme pH (Gomez and Steiner, 2004).

The growth, characterization and identification of thermophiles pose specific problems (Savas *et al.*, 2009). Development of molecular techniques such as PCR-based techniques like rep-PCR and 16S rRNA allow their reliable identification, whereas conventional methods are time consuming and not reliable (Kublanov *et al.*, 2009).

Worldwide, geothermal areas which are favorable habitats for thermophilic organisms are limited to a restrict number of sites. In Morocco, there are more than twenty hot springs distributed in different regions mainly in the Rif, the Pre-Rif and the Southern Rif (Lakhdar *et al.*, 2006). The central region includes the hot springs of Ain Allah and Moulay Yaacoub (Fez region) and Ain Jerri (Meknes region) (Salame *et al.*, 2013). On the South side of the Western Anti-Atlas, the Abaynou spring is located in the middle of an oasis of palms and olive trees. It is considered as the most important hot spring in South Morocco. These springs are frequently visited by Moroccans with dermal and rheumatic diseases (Salame *et al.*, 2013). Merzouga, a small Saharan village, in South Eastern Morocco, is known for its set of sand dunes near Erg Chebbi, which is part of touristic itineraries. The climate is very hot and dry and the temperatures are often higher than 40 °C in the day while nights are cold. In these conditions living organisms have to cope with extremes temperature, low humidity and low availability of nutritional compounds. These conditions reduce biodiversity but some bacteria developed survival strategies in order to adapt to such stress (Hecker and Völker, 2001; Bar *et al.*, 2002).

The wetland of Sidi Moussa-Oualidia is a protected ecosystem of 10.000 ha recognized by the Ramsar Convention of 2005. The complex consists of the lagoons Oualidia and Sidi Moussa and 4 salt marshes (Sidi Abed is one of them). The main activities in the neighborhood of the site are agriculture, livestock raising, sandpit exploitation, salt production, and tourism. Salt mining has reduced biodiversity, especially invertebrates (http://www.ramsar.org).

Little is known about the occurrence and distribution of thermophilic bacteria in Morocco. This work is the first study focusing on culturable thermophilic bacteria: their isolation, identification and characterization. Three kind of sites have been selected for this study *i.e.* hot springs, hot desert soils and salty wetland.

## Materials and Methods

### Sampling sites

**Figure 1:**
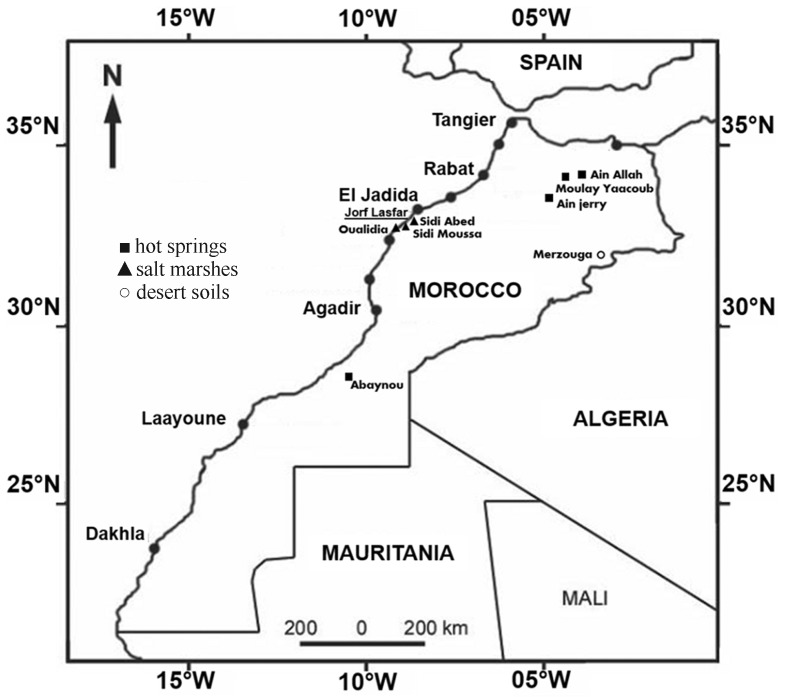
Location of the studied sites.

The location of the sampling sites is shown in Figure 1 and Table 1. Their physico-chemical parameters are given in Table 1. Thirty three samples were collected in sterile conditions, then were transported to the laboratory, using thermal boxes, and immediately analyzed (Malkawi and Al-Omari, 2010).

**Table 1:** Physico-chemical parameters in the sampling sites.

Biotopes	Sampling sites	T (°C)	pH	% NaCl (w/v)	Geographical references
Hot springs	Ain Allah	45	7.3	-	34° 2′19.76″ N
					5° 8′13.18″ O
	Abaynou	39	7.4	-	29°5′26″ N
					10°1′27″ W
	Moulay Yaacoub	57	7	-	34° 5′ 16″ N
					5° 10′ 52″ O
	Ain Jerri	39	7.2	-	33°50′30.28″ N
					5°40′2.41″ O
Salt Marshes	Sidi Moussa	19 to 22	7.6 to 7.7	7.1 to 9	33°01′ N
					08°44′ W
	Sidi Abed	16.5 to 18	8.2 to 8.4	3.2 to 10.8	33°02′ N
					08°42′ W
	Oualidia	18.5 to 20	7.9 to 8.2	1.9 to 3.8	32°46′ N
					09°01′ W
Desert soil	Merzouga	34 to 47	-	-	31° 05′ 57″ N
					4° 00′ 42″ O

Temperature and pH of the sites were measured during sampling. The total salt concentration was determined by titration using Mohr's method (Skoog *et al.*, 1996).

### Enumeration and isolation of thermophilic bacteria

#### Sand samples

An amount of 15 g of each sand sample was homogenized in 15 mL of sterile saline solution (0.9% NaCl (w/v)). Tenfold dilutions were prepared using sterile saline solution. From each dilution, 100 μL were plated on Tryptone Soy Agar (TSA, Difco, Detroit, USA) and incubated at 55 °C for 96 h. The assay was done in triplicate.

#### Water samples

For each water sample, 100 mL were filtered through membrane filters (0.22 μm Millipore Corporation, Bedford); the filters were placed onto TSA and incubated at 55 °C for 96 h in triplicate under aerobic conditions (Maugeri *et al.*, 2001; Malkawi and Al-Omari, 2010).

Bacterial colonies with different morphologies were picked up and plated on TSA until pure cultures were obtained. Numbers were expressed as colony forming units (cfu) (Khiyami *et al.*, 2012). Purified colonies were cultivated on Tryptone Soy Broth (TSB, Difco, Detroit, USA), stored in 20% of glycerol at −80 °C, freeze-dried for further studies and deposited in the Moroccan Coordinated Collections of Microorganisms (www.ccmm.ma).

### Phenotypic study

Characterization of each isolate was performed by observation of its colony morphology, color, size, elevation, margin and Gram and malachite green spore staining. Presence of catalase and oxidase were investigated according to the methods described by Prescott *et al.* (2003).

The ability to grow at different temperatures was evaluated by plating onto TSA and incubating at 55, 60, 65, 70, 75 and 80 °C for 48 h. Halotolerance was assayed by plating each culture onto TSA supplemented with NaCl to total concentrations of 0.5 to 15% (w/v) and incubating at 55 °C for 48 h. Growth was determined by visual observation and all tests were made in triplicate.

Proteolytic and cellulolytic activities were screened qualitatively as described by Sadfi-Zouaoui *et al.* (2008). Amylolytic activity was tested according to Cowan (1991). Lipolytic and cellulolytic activities were revealed according to Sierra (1957). The screening was performed in triplicate.

### Genotypic study

BOXA1R-PCR has been used to cluster all obtained thermophilic isolates. Representative isolates of each cluster were subjected to 16S rRNA gene sequencing.

### DNA extraction and BOXA1R-PCR fingerprinting

Total DNA extraction was performed as described by Pitcher *et al.* (1989). DNA primers corresponding to BOX elements sequences were used as described by Versalovic *et al.* (1994). The PCR products were separated by electrophoresis on 1.5% agarose gels at 35 V for 18 h at 4 °C. BOXA1R-PCR gels stained were visualized under ultraviolet light, followed by digital image capturing using a CCD camera (CCD Camera 570 LTV-Gel SMART, France). These fingerprints were analyzed using BioNumerics software package v6.6 (Applied Maths, Sint Martens Latem, Belgium). Similarity among the digitized profiles was calculated using the Pearson correlation coefficient, and an average linkage (UPGMA) dendrogram was derived (Cherif *et al.*, 2002; De Clerck and De Vos, 2004).

### 16S rRNA gene sequencing and phylogenetic analysis

A set of 144 representative isolates were selected for 16S rRNA gene sequencing using the primers fD1 (5′-AGAGTTTGATCCTGGCTCAG-3′) and rP2 (5′-TACGG CTACCTTGTTACGACTT- 3′). Cleaned PCR products were used as a template for the cycle sequencing reaction (Berrada *et al.*, 2012).

Forward and reverse sequencing were performed using Big Dye Terminator version 3.1 cycle sequencing kit (Applied Biosystems, Foster City, CA) according to the manufacturer's instructions. Sequences assembled were checked and corrected and a preliminary identification was performed by FASTA search of the NCBI database. For a more precise identification, the 16S rRNA sequences were also compared with the prokaryotic small subunit rRNA sequence of the Ez-Taxon database (Kim *et al.*, 2012). The Type and reference strains with highest similarity to the sequences of the studied isolates were retrieved and introduced in BioNumerics database. 16S rRNA sequences were aligned and compared to each other using BioNumerics, and a phylogenetic tree was constructed using the UPGMA algorithm. Isolates were regarded as belonging to a species when sequence similarity with the species type strain was at least 99% and to a genus when sequence similarity with a type strain was at least 97% (Berrada *et al.*, 2012). The 16S rRNA gene sequences, determined in this study, have been deposited in the Genbank database under the accession numbers KF879189 - KF879333.

## Results

### Sampling sites

Eight sites were prospected in Morocco between March 2009 and July 2010: 4 hot springs (Ain Allah, Moulay Yaacoub, Ain Jerri and Abaynou), 3 salt marshes (Sidi Moussa, Sidi Abed and Oualidia), and the desert soils of Merzouga (Figure 1). Temperature values were between 34 and 47 °C in the desert soils, while in hot springs they were between 39 and 57 °C. For salt marshes they were between 16.5 and 22 °C. In hot springs pH values were between 7.1 and 7.4 and in salt marshes they were between 7.6 and 8.4. The average salt concentration varied in salt marshes from 1.9% NaCl (w/v) in Oualidia to 10.8% (w/v) in Sidi Abed (Table 1).

### Thermophilic isolates

The colony forming unit of thermophilic bacteria that grew on TSA at 55 °C, varied from 0.5 × 10^2^ to 1.2 × 10^3^ cfu mL^−1^ in hot springs, from 1.1 × 10^2^ to 1.0 × 10^3^ cfu mL^−1^ in salt marshes and was around 5 × 10^3^ cfu g^−1^ in desert. A set of 240 isolates was obtained: 108 isolates from desert (45%), 79 from hot springs (33%) and 53 from salt marshes (22%) (Table 2).

**Table 2:** Bacteria count and number of isolates in the sampling sites.

Biotopes	Sampling sites	cfu/l or cfu/g	Number of isolates	% of thermophilic isolates
Hot springs	Ain Allah	6 10^2^	31	12.9%
	Abaynou	1.2 10^3^	30	12.5%
	Moulay Yaacoub	0.5 10^2^	5	2%
	Ain Jerri	10^3^	13	5.41%
Salt Marshes	Sidi Moussa	10^3^	16	6.66%
	Sidi Abed	4 10^2^	27	11.25%
	Oualidia	1.1 10^2^	10	4.16%
Desert soil	Merzouga	5 10^3^	108	45%

Of the 240 isolates, 235 (97.9%) were beige-colored, 3 were white and 2 were yellow on TSA. All of the isolates were thermophilic (optimal growth up to 55 °C), Gram positive, rod-shaped, endospore forming and halotolerant (grew between 0.5% and 10% NaCl (w/v), 225 isolates (93.75%) were catalase and oxidase positive.

### BOXA1R-PCR fingerprinting

**Figure 2:**
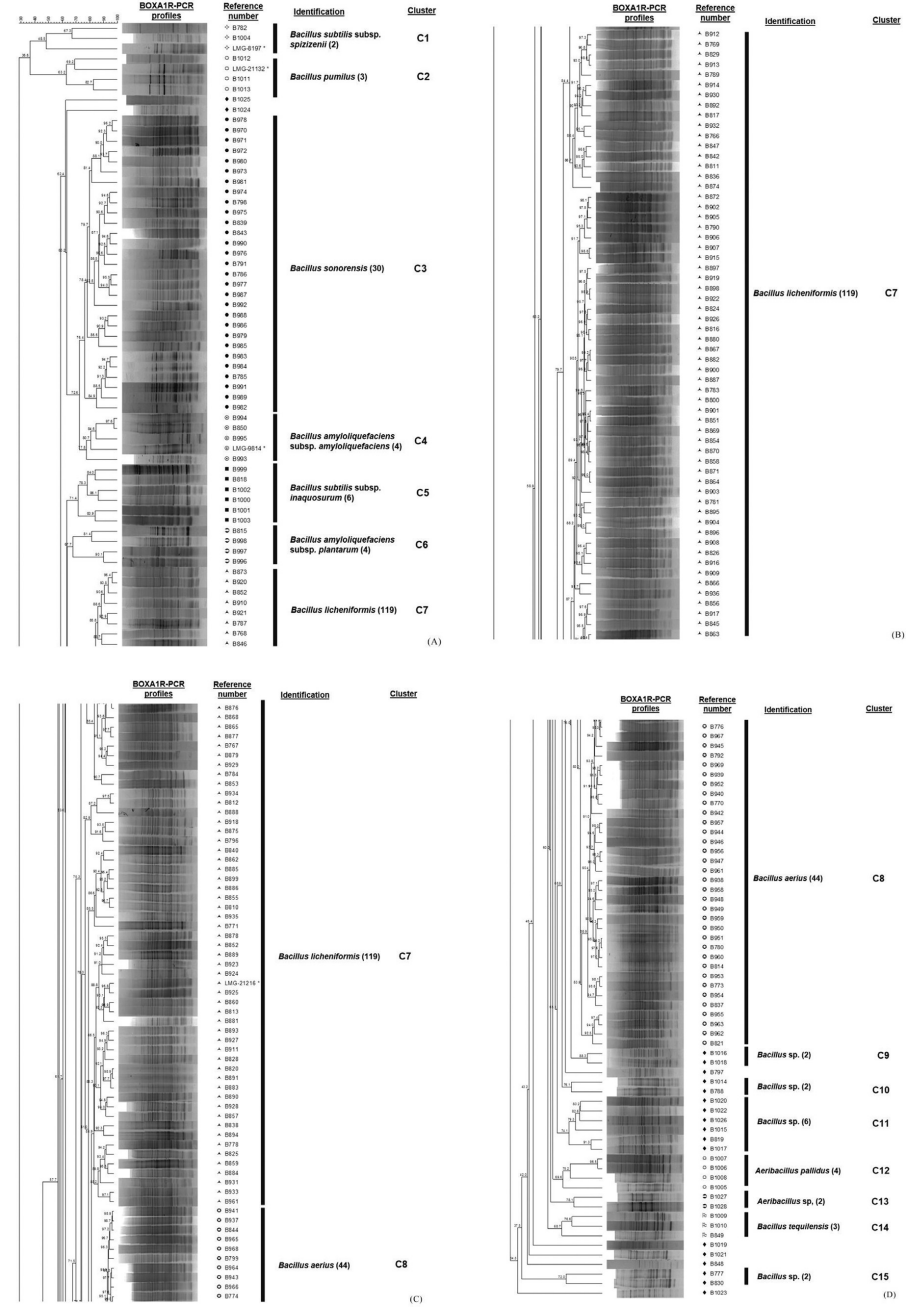
Cluster analysis of BOXA1R-PCR fingerprints and 16S gene sequencing of thermophilic bacteria strains. The dendrogram was generated by the UPGMA method and Pearson correlation coefficient. LMG strains are reference strains used for identification * = reference strain.

The dendogram derived from the BOXA1R-PCR fingerprints (Figure 2) showed 15 clusters (numbered from C1 to C15). Cluster analysis revealed a complex set of relatedness of BOXA1R-PCR patterns among the thermophilic isolates belonging to the same cluster, suggesting a rich population diversity. Only 53 isolates were identified by BOXA1R-PCR when the profiles were compared to those of the reference and type strains available in the CCMM database: 44 isolates were identified as *B. licheniformis* (cluster 7), 4 isolates as *B. amyloliquefaciens* subsp*. amyloliquefaciens* (cluster 4), 3 isolates as *B. pumilus* (cluster 2) and 2 isolates were identified as *B. subtilis* subsp*. spizizenii* (cluster 1) (Figure 2). This identification was confirmed by further 16S rRNA analysis.

### 16S rRNA gene sequencing analysis

In the second step of the genotypic identification, 144 representatives of the 15 clusters and ungrouped isolates were subjected to total 16S rRNA gene sequencing. Sequence analysis of 119 isolates showed a high similarity (≥ 99%) with those of the reference strains available in the Genbank and Ez-taxon databases. Moreover, 21 isolates showed moderate sequence similarity (97 to 98.9%) (Table 3) indicating that they may be potential new species.

**Table 3:** 16S gene sequencing data for the identified strains to the genus level compared to the type strains.

CCMM N°	% of similarity	Reference strains
B777, B788, B830, B848 B1014, B1020, B1022, B1023, B1024 and B1025	97.82 to 98.93	*Bacillus sonorensis DSM 13779*
B797, B819, B1015, B1016, B1017 and B1026	98.42 to 98.99	*Bacillus licheniformis ATCC 14580*
B1019 and B1021	97.95 to 98.37	*Bacillus subtilis* subsp*. inaquosorum NRRL B-23052*
B1018	97.58	*Bacillus aerius JCM 13348*
B1027 and B1028	97.31 to 97.71	*Aeribacillus pallidus ATCC 51176*

The combination of the BOXA1R-PCR and 16S rRNA gene sequencing allowed identifying 219 isolates to the species level. The results revealed a clear domination of the genus *Bacillus* represented by 234 isolates *i.e. B. licheniformis, B. aerius, B. sonorensis, B. subtilis* (subsp*. spizizenii* and subsp*. inaquosurum*), *B. amyloliquefaciens* (subsp*. amyloliquefaciens* and subsp*. plantarum*), *B. tequilensis, B. pumilus* and *Bacillus* sp. In addition, 6 isolates belonged to the genus *Aeribacillus* i.e. *A. pallidus* and *Aeribacillus* sp. (Figure 2, Table 4). The repartition of the obtained species is presented in Table 4.

**Table 4:** Biotope, sampling site and distribution of thermophilic bacteria.

Identified species	Number of strains (%)	Distribution per origin							

		Merzouga	Sidi Abed	Sidi Moussa	Oualidia	Abaynou	Ain Allah	Ain Jerri	Moulay Yaacoub
*B. licheniformis*	119 (49.58%)	49	15	10	7	9	14	10	5
*B. aerius*	44 (18.33%)	23	5	4	3	7	2		
*B. sonorensis*	30 (12.5%)	11	4	2	8	4	1		
*B. subtilis*	8 (3.33%)	3	2	2	1				
*B. amyloliquefaciens*	8 (3.33%)	4	1	1	2				
*B. tequilensis*	3 (1.25%)	1	1	1					
*B. pumilus*	3 (1.25%)	1	2						
*A. pallidus*	4 (1.66%)	4							
*Bacillus* sp*.*	19 (7.9%)	15	3	1					
*Aeribacillus* sp*.*	2 (0.83%)	1	1						
Total	240	108	27	16	10	30	31	13	5

Five *Bacillus* species (*B. licheniformis, B. aerius, B. sonorensis, B. subtilis* and *B. amyloliquefaciens*) were common between hot springs, salt marshes and desert soils (Table 4). A total of 8 species were identified in hot springs and seven in desert soils but *B. licheniformis* was the most abundant species (48.1% and 45.37% respectively). Five species were recovered in salt marshes with *B. licheniformis* as a dominant species (60.4%).

### Effect of temperature

The number of growing strains is reduced between 55 °C and 65 °C, while it's drastically reduced at 70 °C and over. Only 4 isolates tolerate 75 °C and none was able to grow at 80 °C. The number of strains growing at the different temperatures tested are given in Table 5.

**Table 5:** Effect of temperature on growth of different species.

Species	30 °C	55 °C	60 °C	65 °C	70 °C	75 °C	80 °C
*B. licheniformis*	119/119 a	119/119	114/119	72/119	5/119	3/119	0/119
*B. aerius*	44/44	44/44	39/44	26/44	1/44	1/44	0/44
*B. sonorensis*	30/30	30/30	25/30	11/30	1/30	0/30	0/30
*B. subtilis*	8/8	8/8	7/8	5/8	0/8	0/8	0/8
*B. amyloliquefaciens*	8/8	8/8	3/8	2/8	0/8	0/8	0/8
*B. tequilensis*	3/3	3/3	2/3	1/3	0/3	0/3	0/3
*B. pumilus*	3/3	3/3	1/3	0/3	0/3	0/3	0/3
*A. pallidus*	4/4	4/4	4/4	4/4	3/4	0/4	0/4
*Bacillus* sp*.*	19/19	19/19	19/19	4/19	1/19	0/19	0/19
*Aeribacillus* sp*.*	2/2	2/2	2/2	0/2	0/2	0/2	0/2
Total	240	240	216	125	11	4	0

a xi/yi, where xi = number of strains growing at mentioned temperature and yi = total number of strains.

### Effect of salinity

The number of strains growing is reduced with salt concentration increased in the medium. All isolates grew at 3% NaCl (w/v), 220 (91.67%) grew at 5% NaCl (w/v), 183 (76.25%) grew at 10% NaCl (w/v), and none at 15% NaCl (w/v) (Table 6).

**Table 6:** Influence of salinity (%NaCl (w/v)) on growth of different species.

Species	0.5%	3%	5%	10%	15%
*B. licheniformis*	119/119 b	119/119	114/119	97/119	0/119
*B. aerius*	44/44	44/44	40/44	31/44	0/44
*B. sonorensis*	30/30	30/30	26/30	21/30	0/30
*B. subtilis*	8/8	8/8	6/8	6/8	0/8
*B. amyloliquefaciens*	8/8	8/8	7/8	3/8	0/8
*B. tequilensis*	3/3	3/3	2/3	1/3	0/3
*B. pumilus*	3/3	3/3	1/3	1/3	0/3
*A. pallidus*	4/4	4/4	3/4	2/4	0/4
*Bacillus* sp*.*	19/19	19/19	19/19	19/19	0/19
*Aeribacillus* sp*.*	2/2	2/2	2/2	2/2	2/2
Total	240	240	220	183	0

b xi/yi, where xi = number of strains growing at mentioned % NaCl (w/v) and yi = total number of strains.

### Production of thermo-enzymes

Of the 240 strains, 205 (85.42%) produced at least one extracellular hydrolytic enzyme: 171 strains (71.25%) produced amylases, 121 strains (50.41%) produced proteases and only 16 strains (6.67%) produced cellulases. None of the isolates produced lipases. In addition, 9 strains (3.75%) combined the three tested enzymes and 85 strains (35.41%) produced two enzymes (Table 7). 35 strains produced none of the enzymes screened.

**Table 7:** Production of thermo-enzymes.

Species	C1	C2	C3	C4	C5	C6	C7
*B. licheniformis*	37/119 c	17/119	38/119	3/119	0/119	5/119	19/119
*B. aerius*	14/44	6/44	15/44	1/44	1/44	1/44	6/44
*B. sonorensis*	6/30	4/30	12/30	0/30	1/30	2/30	5/30
*B. subtilis*	1/8	2/8	3/8	0/8	0/8	1/8	1/8
*B. amyloliquefaciens*	4/8	0/8	4/8	0/8	0/8	0/8	0/8
*B. tequilensis*	1/3	0/3	2/3	0/3	0/3	0/3	0/3
*B. pumilus*	0/3	2/3	1/3	0/3	0/3	0/3	0/3
*Aeribacillus pallidus*	1/4	0/4	1/4	0/4	0/4	0/4	2/4
*Bacillus* sp*.*	15/19	1/19	1/19	1/19	0/19	0/19	1/19
*Aeribacillus* sp*.*	0/2	0/2	1/2	0/2	0/2	0/2	1/2
Total	79	32	78	5	2	9	35

c xi/yi, where xi = number of strains producing enzymes and yi = total number of strains.

C1: amylases, C2: proteases, C3: amylases and proteases, C4: amylases and cellulases, C5: proteases and cellulases, C6: amylases, proteases and cellulases, C7: No enzymes.

## Discussion

Thermophilic bacteria were present in all samples analyzed. Nevertheless, total counts were very low (50–5000 cfu mL^−1^). In a recent study of thermophilic bacteria in ten Saudi Arabia hot springs, Khiyami *et al.* (2012) reported the same order of magnitude (170 – 1320 cfu mL^−1^). Despite all of the recovered strains can grow at 30 °C, but their optimal growth temperature is 55 °C (temperature of isolation). Thus, all strains could be classified as thermophilic by Brock (1978), Souza and Martins (2001) and Cihan *et al.* (2012). Based on the optimal concentration of salt and according to Kushner *et al.* (1985), all the isolated strains were halotolerants. Morphologic, physiologic and microscopic characteristics of recovered isolates were consistent with the description of the genus *Bacillus*, according to Gordon *et al.* (1973) and Souza and Martins (2001). High number of isolates obtained in desert (108 isolates) and hot springs (79 isolates) could be explained by the hot conditions (temperature ranging from 34 °C to 57 °C) in these biotopes exerting a pressure for the selection of thermophilic flora mostly while in salt marshes characterized by mesophilic conditions (temperature ranging from 16.5 °C to 22 °C) only 53 isolates were recovered.

Based on BOXA1R-PCR or/and 16S rRNA sequence analysis, 97.5% of isolates were assigned to the genus *Bacillus* and 2.5% to the genus *Aeribacillus*. Their combination allowed identifying 219 isolates (91.25%) to the species level (Figure 2 and Table 4), indicating that these techniques were not only a powerful tool for identification of thermophilic bacteria to the species level but also revealed a considerable intra-species diversity (De Clerck and De Vos, 2004; Adiguzel *et al.*, 2009). Twenty one isolates remained unidentified to the species level; nineteen were assigned to *Bacillus* sp*.* and two to *Aeribacillus* sp*.* This could be an indication for the presence of potential new thermophilic species. These isolates need to be further analyzed.

The dendrogram derived from BOXA1R-PCR profiles showed a high discriminatory level and revealed 2 sub-groups among *B. licheniformis*. This is in agreement with the finding of De Clerck and De Vos (2004). Moreover, Manachini *et al.* (1998) while studying 182 *B. licheniformis* strains reported three distinct groups that were therefore regarded as genomovars of *B. licheniformis.* Our BOXA1R profiles were helpful in distinguishing between sub-species of *B. subtilis* (subsp. *spizizenii* and subsp. *inaquosurum*) and also *B. amyloliquefaciens* (subsp. *amyloliquefaciens* and subsp. *plantarum*). This is in agreement with the grouping reported by Connor *et al.* (2010).

Physiological behavior of the identified strains showed several differences between strains of the same species, such as tolerance to NaCl, temperature and production of hydrolytic thermo-enzymes. This finding is in agreement with previous reports (Burgess *et al.*, 2010; Adiguzel *et al.*, 2011).

### Diversity recovered

The extent of bacterial diversity detected in this study is not surprising as the majority of bacteria found in the investigated biotope occur commonly in the environment and have been described in many different environments studied elsewhere. Strains of the genus *Bacillus* are well adapted to hot and dry environments (Meintanis *et al.*, 2008; Kawasaki *et al.*, 2012). They have also generally simple nutritional needs. Therefore, they do not require specific nutrients for growth and are able to colonize oligotrophic niches like salt marshes, hot springs and desert soils (Cihan *et al.*, 2012; Khiyami *et al.*, 2012). Nevertheless, *B*. *aerius* and *B*. *tequilensis* were reported, for the first time, as thermophilic bacteria in this study.

Malkawi *et al.* (2010), reported that 97% of the recovered thermophilic isolates belong to the genus *Bacillus*. Abou-Shanab (2007) identified 13 thermophilic strains, from several Jordanian hot springs, belonging to the genus *Bacillus* (*B. licheniformis* and *B. pumilus*). In another study, Maugeri *et al.* (2001), isolated 87 thermophilic, aerobic and spore-forming bacteria from Eolian Islands (Italy). Most of them were members of *Bacillus*. Kawasaki *et al.* (2012) isolated 12 thermophilic bacteria belonging to the genus *Bacillus*, from a saline hot spring in Japan. They were also relatively halotolerant (grew in a medium with 10% NaCl (w/v)) which is consistent with our finding. Moreover, Perfumo and Marchant (2010) reported the presence of thermophilic *Aeribacillus* strains in samples of dust collected from Turkey and Greece. The presence of thermophilic strains belonging to *A. pallidus* from hot springs was reported by Chamkha *et al.* (2008) and Savas *et al.* (2009).

*Bacillus licheniformis* was the dominant species (approximately 50% of total strains), which is in agreement with those obtained by Derekova *et al.* (2008). Thermophilic strains of *B. pumilus, B. amyloliquefaciens* and *B. subtilis* have been isolated from hot springs, salt lakes, volcanic area in Turkey, Bulgaria, Iceland, Jordan, Egypt (Meintanis *et al.*, 2008; Minana-Galbis *et al.*, 2010 ; Cihan *et al.*, 2012). Ngoc-Phuc *et al.* (2007) reported the dominance of *B. licheniformis* and also the presence of *B. subtilis* in a Japanese desert. Lester *et al.* (2007) reported the presence of *B. subtilis* and *B. pumilus* from extreme arid Atacama Desert soils. In Morocco, Berrada *et al.* (2012), showed that salt marshes in the north of Morocco are colonized by a number of halo-thermophilic bacteria belonging especially to *B. pumilus* and *B. licheniformis. Bacillus sonorensis* is commonly observed in arid areas, such as the Sahara, Mojave, Sonoran, and Gobi Deserts (Palmisano *et al.*, 2001).

### Distribution of thermophilic bacteria

The genus *Bacillus* was isolated from the 8 explored biotopes and the genus *Aeribacillus* was obtained only in the hot spring of Abaynou and desert of Merzouga. The occurrence of *Bacillus* strains in all investigated sites could be explained by the fact that *Bacillus* has been shown to migrate at extremely high rates, even between continents, and because *Bacillus* spores are known to resist to the environmental stress (Connor *et al.*, 2010). *B. licheniformis* was isolated from all sites and was also the unique species identified in the site of Moulay Yaacoub and Oualidia. *B. licheniformis* is an ubiquitously occurring spore-forming bacterium widely distributed as a saprophytic organism in the environment (Manachini *et al.*, 1998; Rey *et al.*, 2004).

The diversity obtained from the sand of Merzouga and the hot spring of Abaynou was very close (both located in a Saharan area) which could be explained by the perpetual movement of sand particles shipped with the wind and carried microorganisms attached thereto (Perfumo and Marchant, 2010). The low diversity detected in Moulay Yaacoub (only 5 strains belonging to *B. licheniformis*) could be explained by the high temperature (57 °C) and mineralization and also the oligotrophic statute of this hot spring (Lakhdar *et al.*, 2006; Salame *et al.*, 2013). Tekere *et al.* (2012), recovered a few bacteria phylotypes at Tshipise hot spring, where high temperature (58 °C) and high dissolved mineral salts occurred, which is consistent with our finding. It could be also explained by their composition in gases dominated by nitrogen, methane and carbon dioxide. Moreover, it has been reported that oxygen is only present in traces (Lakhdar *et al.*, 2006). This could inhibit the development of aerobic flora (*Bacillus*, *Aeribacillus*, *Geobacillus*) and promotes more probably anaerobic flora (*Clostridium, Anaerobaculum, Thermoanaerobacter, Thermoanaerobacterium*) (Robb *et al.*, 2008).

A total of five identified species belonging to the genus *Bacillus* were recovered in all the three salt marshes while three other isolates remain unidentified. This relatively low diversity could be explained by the negative effect of high content of salt in these sites resulting in reducing biodiversity (Ventosa *et al.*, 1998). The three sites are also situated in a Moroccan region with intense agricultural activity. The use of chemical pesticides could reduce diversity of all organisms mainly bacteria. The number of different species obtained from Oualidia was less than those from Sidi Abed and Sidi Moussa. The site of Oualidia (located in direct contact with marine water) is influenced by the quality of marine water with increasing quantities of heavy metals (Pb, Cd, Zn, Cr, As). Atlantic Moroccan coast is characterized by lead contamination that occurs around areas receiving industrial waste outflows, mainly zone situated near the discharge from phosphate transformation industries located at Jorf Lasfar (region of El Jadida) which is very close to the marshes sampling sites (Figure 1) (Benbrahim *et al.*, 2006).

### Production of thermo-enzymes

It has been reported that thermophilic bacteria, mainly *Bacillus* strains, produced high valuable thermo-enzymes (Meintanis *et al.*, 2008). In accordance with their saprophytic life style, the secretome of *Bacillus* strains encodes numerous secreted enzymes that hydrolyze polysaccharides, proteins, lipids and other nutrients (Rey *et al.*, 2004). Strains obtained in this study produced an assortment of extracellular enzymes that may contribute to nutrient cycling in nature (Rey *et al.*, 2004). In this work, 70.83% and 50.41% of total strains recovered exhibited high amylolytic and proteolytic activity respectively. While, only 19 strains (5.41%) produced cellulases. In addition, 9 strains (3.75%) belonging to *B. licheniformis* (5), *B. aerius* (1), *B. sonorensis* (2) and *B. subtilis* (1) produced all the extracellular hydrolytic enzymes screened except lipase. This finding indicated also that the tested strains may have developed genetic and physiological capability for utilizing available organic matter, via exo-enzymes production (Berrada *et al.*, 2012). It could be also explained by trend of microbial societies toward surviving at low organic content in such niches and development of adaptable system for uptake of any available food (Derekova *et al.*, 2008).

## Conclusion

This is the first investigation of thermophilic bacteria in Moroccan hot springs, salt marshes and desert. The results showed a clear domination of thermophilic *Bacillus* species mainly *B. licheniformis*. These strains might be a source of industrially important enzymes mainly amylases and proteases.
